# Association of Fig Pollinating Wasps and Fig Nematodes inside Male and Female Figs of a Dioecious Fig Tree in Sumatra, Indonesia

**DOI:** 10.3390/insects13040320

**Published:** 2022-03-24

**Authors:** Jauharlina, Hartati Oktarina, Rina Sriwati, Muhammad Sayuthi, Natsumi Kanzaki, Rupert J. Quinnell, Stephen G. Compton

**Affiliations:** 1 Department of Plant Protection, Agriculture Faculty, Universitas Syiah Kuala, Banda Aceh 23111, Indonesia; oktarina.hartati@unsyiah.ac.id (H.O.); rinasriwati@unsyiah.ac.id (R.S.); say_m2001@unsyiah.ac.id (M.S.); 2 Kansai Research Center, Forestry and Forest Products Research Institute, 68 Nagaikyutaroh, Momoyama, Fushimi, Kyoto 612-0855, Japan; nkanzaki@affrc.go.jp; 3 School of Biology, Faculty of Biological Sciences, University of Leeds, Leeds LS2 9JT, UK; r.j.quinnell@leeds.ac.uk (R.J.Q.); s.g.a.compton@leeds.ac.uk (S.G.C.)

**Keywords:** Agaonidae, *Caenorhabditis*, dioecy, *Ficus*, phoresy, vector

## Abstract

**Simple Summary:**

It has been known that fig pollinating wasps can transfer nematodes when they enter receptive figs to deposit their eggs (male figs in male trees) and pollinate the flowers (female figs in female trees) of a dioecious fig tree. However, the fate of nematodes transferred into female figs was unknown, since the pollinating wasps that enter female figs cannot reproduce. This study examined whether the nematodes transferred by pollinating wasps into female figs of *Ficus hispida* could develop and reproduce in the same way as in male figs. Three species of nematodes were found to develop within the male and female figs of *F. hispida*, with populations peaking at about the same time for both sexes of figs. Our findings showed that the female figs could support the growth and reproduction of the three nematodes; however, there was no pollinator offspring to transfer the nematodes out of the figs. The results provide a better understanding of the development of nematodes in male and female figs of a dioecious fig tree, which may also influence the biodiversity of the tropical ecosystem.

**Abstract:**

Nematodes can grow within the inflorescences of many fig trees (*Ficus* spp., Moraceae); however, the feeding behaviour of most nematodes is not known. Fig pollinating wasps (Hymenoptera: Agaonidae) transfer nematodes into young figs upon the wasps’ entry into the figs to deposit their eggs. Most Asian fig trees, however, are functionally dioecious, and the pollinating wasps that enter female figs are unable to reproduce. They fail to produce the offspring required to carry the new generations of nematodes. We examined whether female figs of *F. hispida* can nonetheless support the development of phoretic nematode populations. Nematodes were extracted from male and female figs sampled in Sumatra, Indonesia, to compare the growth of their populations within the figs. We found three species of nematodes that grew within figs of male and female trees of *F. hispida*: *Ficophagus* cf. *centerae* (Aphelenchoididae), *Martininema baculum* (Aphelenchoididae) and *Caenorhabditis* sp (Rhabditidae). The latter species (*Caenorhabditis* sp.) has never been reported to be associated with *F. hispida* before. Nematode populations peaked at around 120–140 individuals in both sexes of figs, at the time when a succeeding generation of adult fig wasps appeared within male figs. The female figs could support the growth and reproduction of the three nematodes species; however, the absence of vectors meant that female figs remained as traps from which there could be no escape.

## 1. Introduction

Fig trees (*Ficus* spp. family Moraceae) are a large and diverse group of mainly tropical and sub-tropical trees. Their inflorescences (figs, also called syconia) are characterised by their unusual enclosed structure. Ripe mature figs are important resources for frugivorous vertebrates [1], but before figs ripen, they support complex insect communities that include fig wasps, flies, beetles and moths [2]. Other invertebrates, such as mites and nematodes, also colonise the figs [3,4,5], together with microorganisms such as fungi and protistans [6].

The development of a fig usually depends on it being entered by female pollinating fig wasps (Chalcidoidea, Agaonidae). They go into figs to deposit eggs in the ovules lining the internal part of the figs [6]. Their larvae grow individually within the ovules, which are galled by the female wasps when eggs are deposited [7]. After they enter, the fig wasps also actively or passively pollinate some of the flowers [8]. The pollinators lose their wings on entry, so they cannot enter figs on more than one tree, and many of them do not reappear from the first fig into which they enter [9]. Some non-pollinating fig wasps (belonging to several families of Chalcidoidea) can also stimulate fig development, but most of them lay eggs from outside of the figs by inserting their ovipositor through the fig wall. This is significant for the broader community of animals that develop inside figs, because the pollinator females that enter figs bring with them phoretic organisms such as mites and nematodes [4,10,11].

Fig trees show two distinct breeding systems that differ in their value to the animals using fig wasps for transport. Individual figs in monoecious fig tree species have both male and female flowers, and the development of both seeds and pollinating fig wasps occur in female flowers. Some individuals of dioecious fig tree species produce functionally male figs that aid in the growth of a new generation of fig wasps and pollen, while others possess female figs and reproduce through seeds [12,13]. Pollinators are unable to differentiate male and female figs and are attracted to figs of both sexes. Therefore, any phoretic species transferred between figs of a dioecious fig tree are routinely taken into a female fig, from which no fig wasp offspring will serve as vectors. Female trees often concentrate their fruiting during certain seasons, and the majority of the figs available for pollinators to enter will be female. This significant cause of mortality means that phoretic mites are almost exclusively associated with monoecious fig tree species [4]. On the contrary, phoretic nematodes have been found from figs of both monoecious and dioecious fig tree species [14].

According to Dominican amber fossils, pollinating fig wasps have transported nematodes between figs for at least twenty million years [15]. Today, nematodes have been found in fig trees throughout their entire distributional and taxonomic range [16,17,18,19]. The nematodes are classified into several different families, implying multiple independent fig colonisations accompanied by widespread radiation in some lineages [20]. Nematodes from several different species can grow and develop in one fig tree species, and it has been found that in Indonesia, *F. racemosa* can support up to eight species of nematodes [14], with more than one species sometimes present inside a single fig. The figs support nematodes to grow and reproduce offspring that are ready to be transported by a new generation of adult female pollinators that come out of their natal figs [3,10]. Nematodes that wait to adhere to female pollinating wasps may be strewn near the interior of the fig or clumped together in male flowers if their fig wasp vectors display active pollen-collecting behaviour [8,10].

Most fig nematode feeding requirements are undetermined, but they are obviously diverse [21,22]. Plant-feeding nematodes are shown by the presence of stylets, but different species may have different preferred feeding areas inside the figs [21]. The stylets are used to pierce plant cells, extract food and emit proteins and metabolites that help the nematodes while they eat [23]. Several species of nematodes that have no stylet are known to consume the rotting bodies of pollinator females, and some of them may even start to feed on the females prior to their death [24]. During their developments, figs from the subgenus *Sycomorus* are often partially filled with liquid [25], and they have diverse nematode fauna. These free-swimming nematodes can prey on other nematodes or protistans found abundantly in the fig liquid [14].

The male and female syconia of *Sycomorus* figs provide some potential resources for nematodes that are common to both sexes, but there are also differences. Figs of both plant sexes will contain the decaying bodies of pollinators, and both can fill with liquid; however, only figs from male trees have male flowers and pollen. Female flowers are present in figs from both male and female trees, but their floral development differs, resulting in either galled ovules or seeds. Here, we address the following questions about the nematode species associated with *F. hispida*, a dioecious fig tree species grown in Sumatra, Indonesia: (i) How many nematode species are present? (ii) How large are their populations? (iii) Are they able to grow and reproduce within figs from both male and female trees?

## 2. Materials and Methods

### 2.1. Study Species and Site

The fig tree species *Ficus hispida* is a widely distributed dioecious shrub or small tree [26]. The figs are produced mainly on leafless branches and the trunks and are often at different phases of development on the same tree [27]. The C phase figs of *F. hispida*, like many other fig tree species in the *Sycomorus* subgenus, frequently contain noticeable quantities of liquid [26]. In Indonesia, *Ceratosolen solmsi marchali* is the only pollinating wasp found on *F. hispida* [21]. However, the tree is home to a variety of *Ceratosolen* pollinators in other areas. *Ficus hispida* is known to support a variety of non-pollinating fig wasp species, but none of these enter the figs to oviposit, and they are unlikely vectors of nematodes.

Development of fig phases was documented as described previously in earlier studies [21,28]. Figs in phase A are pre-receptive. Phase B figs are at the stage when female pollinating wasps (and nematodes) enter through ostioles. During the C phase, a new generation of fig wasps (in male fig trees) and seeds (in female fig trees) grow and mature, as do any nematodes that reproduce within the figs. In D phase figs, the newly emerged male pollinating wasps copulate with female wasps that are still inside their galls. The female pollinating wasps then depart from their galls, deliberately gather pollen, and store it into pollen baskets. The female wasps then come out via an escape hole cut by the males in the fig wall. Soon after that, the E phase starts. There are no D phase figs in female trees. The female figs ripen once the seeds inside reach maturity, and they attract dispersers such as frugivorous vertebrates to consume (E phase).

Figs of *F. hispida* were observed in Aceh Province, the northern part of Sumatra, Indonesia. *Ficus hispida* trees are common as roadside trees in the area, usually forming clumps of several trees in which male and female trees coexist. The area has a tropical climate, with relatively stable annual average temperatures but highly variable rainfall. During the period of study, the average daily temperature was 28.22 °C ± 0.09 °C (mean ± SE) (range 25 °C–32 °C), with an average minimum of 24.18°C ± 0.09°C (range 22 °C–28 °C) and a maximum of 32.26 °C ± 0.15 °C (range 28 °C–35 °C). During the study, March was the driest month, having no rain, while May, with 15 days of rain (361.7 mm), was the wettest month.

Fig cohorts were sampled from fig wasp entry through to the emergence of fig wasp offspring from male figs and the ripening of female figs. Samples were removed at weekly intervals from six trees (three males, three females) for a period of six months from March 2018. The fig trees grew alongside public roads in the Leupung District’s mountainous region, at 05°22′55″ N and 95°15′34″ E, about 25 km from the city of Banda Aceh. Figs were present more or less continuously on *F. hispida* trees of both sexes. Asynchronous fig fruiting often resulted in the presence of different developmental stages at any given time; however, some trees had relatively discrete ‘cohorts’ that enabled the growth of certain groups of figs on each tree to be tracked. Sampling was conducted to follow each cohort’s development.

### 2.2. Sampling Methods and Fig Extraction

The development times of marked fig cohorts were determined from the appearance of the first A phase figs to the formation of the first E phase figs. A random sample of ten figs was taken from each cohort of each tree from A to E phases. Later the same day as the figs were collected, they were opened, and the numbers of dead adult fig wasps present inside any B phase figs was recorded. Five to six figs from each tree, including their contents, were then placed separately in a Baermann extraction funnel using a method described previously [29] and adjusted later [14]. Each fig was later cut into eight pieces and set on a fine fabric sheet before being submerged in 60 mL distilled water. The funnel’s water content ensured that the fig fragments remained immersed. After 24 h, the liquid under the funnel was transferred to 20 mL reaction tubes and allowed to stand alone for 3 h. The upper 15 mL of liquid contained no nematodes and was removed using a small pipette. Five one-ml extracts from the remaining five millilitres in the tubes’ bottoms were placed on a one-ml capacity nemacytometer glass slide (counting slide) and observed under a microscope. The total numbers of nematodes present in each subsample were recorded, and the numbers of adults of each species were also distinguished (juvenile nematodes could not be identified further).

### 2.3. Statistical Analysis

The effects of tree sex on (i) number of pollinators entering a B phase fig, (ii) number of nematodes inside B phase figs, and (iii) number of nematodes in D phase figs were analysed by generalised linear models with Poisson errors using the glmer package in R version 3.3.1 [30]. Linear mixed effect models were applied with the tree identity included in the model as a random effect. The different size of figs between male and female trees was tested using a t-test with unequal variance assumption. 

## 3. Results

### 3.1. Fruiting Phenology and Fig Wasp Entrants

The development times of 430 figs were recorded. On male trees, the period of fig development from A to E phases took place for seven to eight weeks and similarly, on female trees, it lasted for seven weeks. The number of female pollinating wasps that entered figs at the B phase varied between one and three, with an average of 1.30 ± 0.1 per fig (Mean ± SE, N = 30 figs) on male trees and 1.27 ± 0.1 per fig (Mean ± SE, N = 30 figs) on female trees. There was no difference in the number of fig wasp entries between male and female trees of *F. hispida* (glmer, z = 0.11, *p* = 0.90).

### 3.2. Fig Nematodes and Their Life Cycles

**Figure 1:**
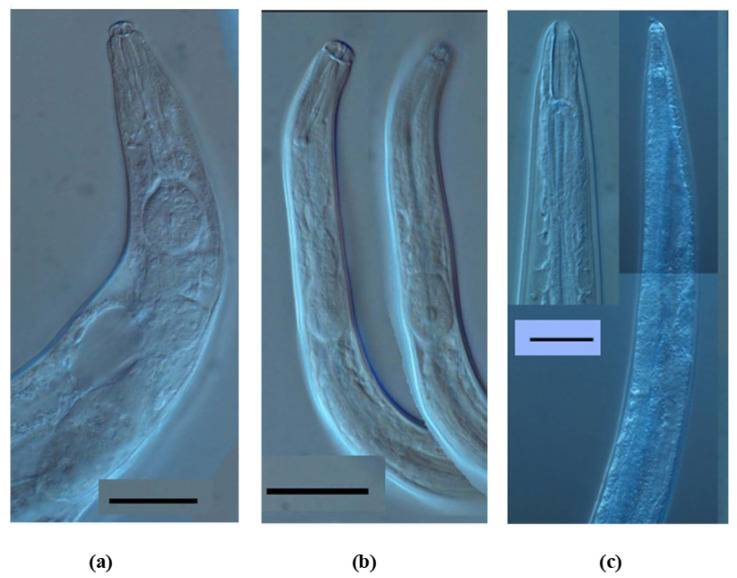
Diagnostic features of adults of nematodes species found inside male and female figs of *Ficus hispida*. Adults of *Ficophagus* cf. *centerae* (**a**) *Martininema baculum* (**b**) and *Caenorhabditis* sp (**c**) showing their specific mouthparts. Bars represent 20 μm for (**a**,**b**) and 50 μm for (**c**).

The growth and population trends of nematodes were examined from a sub-sample of 185 figs (91 male figs and 94 female ones) by extracting their contents in the laboratory. Nematodes were found in every fig sample taken from male trees (100% occupancy) and in the majority of fig samples taken from female trees (86/94, 91.5% occupancy). We recorded three nematode species: *Caenorhabditis* sp. (Rhabditidae), a putative bacteria feeder, and two species from the family Aphelencoididae: *Ficophagus* cf. *centerae* and *Martininema baculum*, known as plant parasites. All three species of nematodes were recorded inside figs taken from male and female trees (Figure 1). The identification of *Ficophagus* cf. *centerae* and *M. baculum* were made based on molecular profiles [31]. *Caenorhabditis* sp. is typologically and phylogenetically close to, but distinct from *C. inopinata*, another nematode species associated with fig trees [32,33].

**Figure 2:**
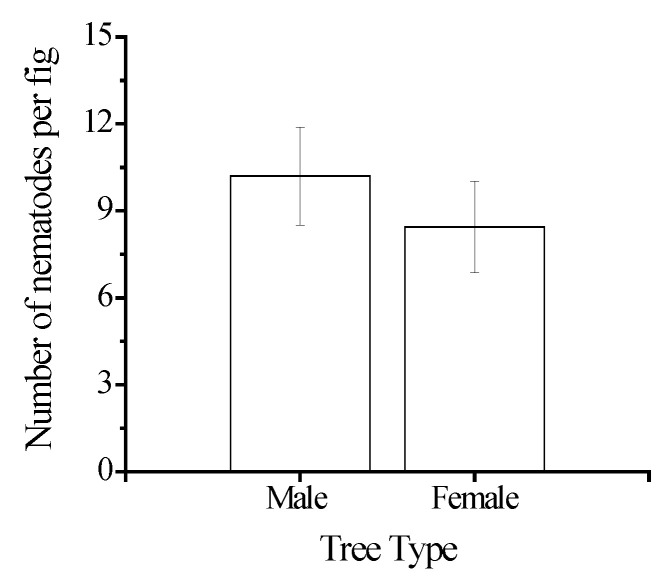
The number of nematodes present inside B phase figs from male and female trees of *Ficus hispida*, soon after the fig wasp vectors of nematodes entered and died (means ± SE, N = 15 figs from male trees, N = 18 figs from female trees).

A sub-sample of smaller figs that foundresses had only recently entered (B phase) contained only pre-adult or juvenile stages of the three nematodes. Recently dead fig wasps in these figs often had one or two nematodes still present on or near them (22 of 30 male figs and 17 of 30 female figs collected from three trees of each sex). However, most nematodes were not attached to their vectors and had dispersed onto the inner surface of the figs. The total number of immature nematodes discovered within newly entered male figs ranged from 3 to 26 (Mean ± SE =10.20 ± 1.68, N = 15 figs) and 0 to 20 in female figs (Mean ± SE = 8.44 ± 1.57, N = 18 figs). The numbers of nematodes in male and female B phase figs did not differ significantly (glmer, z = 0.46, *p* = 0.76; Figure 2). 

**Figure 3:**
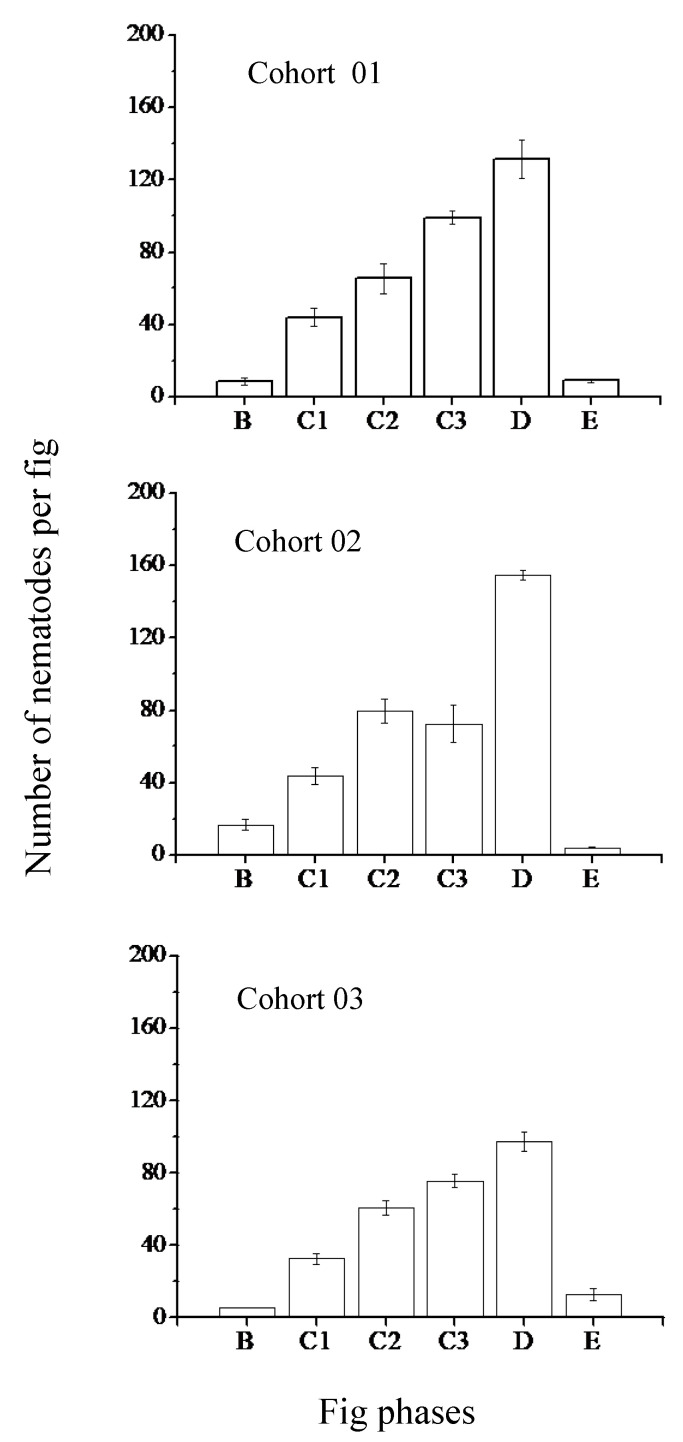
Nematode population sizes during the development of male figs of *Ficus hispida*. All nematode species and stages combined (means ± SE, N = 5 to 6 figs for every phase). Pollinator fig wasp vectors of the nematodes enter during the B phase. Phases C1–C3 cover the early, middle and late periods when fig wasps are developing. The new generation of fig wasps start to emerge from their galls in male figs during the D phase. After the female fig wasps have departed, the males die, the figs start to shrivel and eventually fall from the trees.

**Figure 4:**
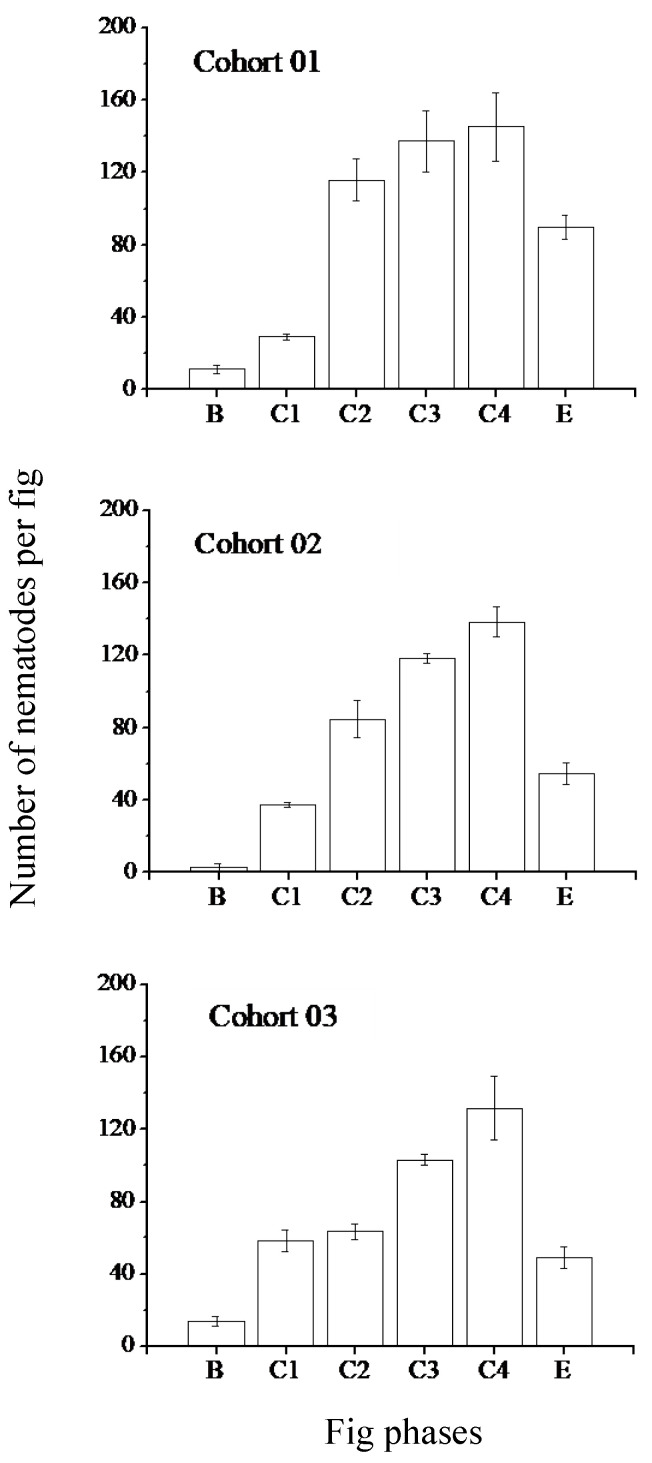
Nematode population sizes during the development of female figs of *Ficus hispida*. All nematode species and stages combined (means ± SE, N = 5 to 8 figs for every phase). Pollinator fig wasp vectors of the nematodes enter during the B phase. Phases C1–C3 cover the early, middle and late periods when seeds are developing. Female figs have no D phase. Phase C4 covers the equivalent period when the seeds mature but before the figs soften, change colour, and become attractive to vertebrates (E phase).

Nematodes continued to be present in the central lumen of the figs throughout fig development. They were also present in the liquid that was present in varying amounts as the figs developed. The three nematode species produced adults of the new generation within both the male and female trees (Figure 3 and Figure 4). Adult and immature nematodes were present in all phases of fig development except the very early phases (A Phase) in both figs from male and female trees. This finding suggests that two or more generations were able to develop in the time that a single generation of pollinator offspring and the seeds were developing. The peak population sizes in male figs were at the D phase, corresponding with the time when adult fig wasps of the new generation were starting to emerge from their galls, ahead or exiting the figs (Figure 3). There were no nematodes recorded from inside the anthers. Female figs from female trees do not contain male flowers. They do not have a direct equivalent developmental phase because no new generation of fig wasps are produced. Still, nematode populations peaked at approximately the same time (late C phase), when the figs started to soften and become attractive to vertebrate frugivores (Figure 4). The peak nematode populations did not differ in male and female figs (glmer, z = −0.837, *p* = 0.40).

**Figure 5:**
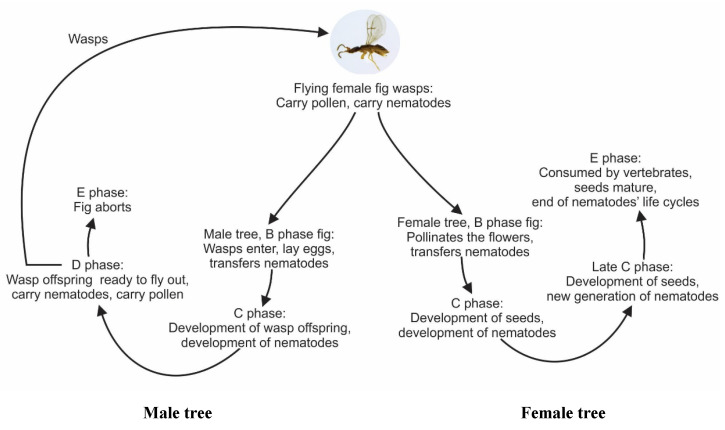
Summary of the life cycles of fig nematodes and pollinator fig wasps associated with male and female *Ficus hispida*.

Opening of mature figs just before the next generation of fig wasps emerged revealed that nematodes developing inside the male figs attached themselves to the female pollinator as soon as the females became available, starting when the males made holes into the females’ galls to copulate. All nematodes that had developed in female figs could only remain there until the figs were eaten or fell to the ground. The life cycles and contrasting fates of the nematodes in the male and female figs are summarised in Figure 5.

## 4. Discussion

The figs from male and female *F. hispida* on the six trees were available for colonisation by fig wasps throughout the study period. The numbers of female pollinators (foundresses) entering B phase figs from male and female trees were similar. This result showed that the female pollinators did not discriminate against figs on female trees, even though they lose their wings when entering these figs, and the figs inhibit their reproduction. The similar numbers of fig wasps entering male and female figs resulted in similar numbers of nematodes being carried into the figs. The nematodes belonged to three species, two of which have adaptations for feeding on plant tissues [31,32]. Adults of all three species were present on the surface of the seeds and galled ovules. They were also present in the liquid that temporarily filled much of the interior of the figs. Small immatures of all three species moved away from the cadavers of the female fig wasps that had brought them into the figs, suggesting that none of the three, including *Caenorhabditis* sp., feed on their cadavers. Likely alternative food sources for *Caenorhabditis* sp. include microorganisms carried into the figs by the fig wasps such as bacteria, yeasts and protistans, as for its close relative, *C. inopinata* [11,34,35]. In the present study, three nematode species were combined mostly because the discrimination was difficult for small juveniles. However, the propagation pattern could be different between plant parasites (*F.* cf. *centerae* and *M. baculum*) and bacteria feeders (*Caenorhabditis* sp.). Further species-level analyses employing molecular markers, etc., will yield the information of more detailed population dynamics of these nematodes. All three nematode species reproduced successfully in both the male and female figs. Adults of the three species were present throughout much of the time the figs were developing, suggesting that several overlapping generations were produced. Resources absent from female figs (galled ovules and male flowers) were obviously unnecessary for the nematodes. Their populations also expanded at similar rates in the male and female figs, suggesting that the figs provided resources of similar quality. However, our sampling could not determine whether populations of all three species had grown at similar rates in male and female figs. 

Inside male figs, males of the new generation of adult fig wasp offspring emerged from their galls before the females. At this time, there was little liquid remaining in the figs. The inner surface of the figs was often covered with hundreds of immature nematodes, some of which took advantage of the mating holes chewed by the males to gain access to the females before they had emerged from their natal galls. Unlike some other nematodes associated with fig wasps that display active pollen collection, the nematodes did not aggregate on the stamens but directly targeted the females [10].

Most research on nematodes in dioecious fig trees has concentrated on male trees that generate new generations of fig wasps [32,36]. The nematode life cycles *in F. hispida* figs were mainly similar to those previously described in male figs of other fig trees of both dioecious and monoecious species [4,14,37,38]. However, our research demonstrated that nematodes associated with *F. hispida* can grow and reproduce within figs from female trees, as shown by a recent study in China [39]. The female figs eventually ripened and became attractive to frugivorous vertebrates. If the figs were not eaten, they eventually fell to the ground and rotted. The continued survival of the nematode populations is therefore not possible. Despite this, the nematodes were almost ubiquitous inside the figs of *F. hispida*.

The abundance of nematodes inside figs of *F. hispida* trees had no discernible effect on the growth of pollinators within the galls inside the figs from male trees or on the development of seeds inside those from female trees. In figs from male trees, pollinator reproductive success is vital for new generations of nematodes to survive, as the decreased reproductive success of pollinators results in fewer vectors for the nematodes’ offspring [24]. Even among the nematode species that eat dead or dying pollinators after entering new figs, there is hardly any indication that nematodes are detrimental to the survival of fig wasps [40]. Thus, nematodes contribute to the exceptional biodiversity of figs without interfering with the essential mutualism that underpins that richness.

## 5. Conclusions

Fig nematodes transferred by fig pollinating wasps into figs have been recorded worldwide, mostly in monoecious figs and male figs of dioecious fig tree species. This study has shown that phoretic nematodes were transported and developed equally in male and female figs of *Ficus hispida*, a dioecious fig tree species commonly grown in Sumatra, Indonesia. We recorded two species of nematodes from the family Aphelencoididae: *Ficophagus* cf. *centerae* and *Martininema baculum*, known as plant parasites, and *Caenorhabditis* sp. (Rhabditidae), a putative bacteria feeder that had never been found in *F. hispida* figs before. The female figs could support the growth and reproduction of the three nematode species. However, there was no means for the nematodes to be transported outside the figs since no new generation of pollinating wasps developed inside the female figs. The female figs remained as traps from which there could be no escape for the nematodes that developed and reproduced inside the figs. This study has shown that while pollinating wasps do not reproduce inside female figs, the nematodes they transfer can grow and reproduce in the same pattern as inside the male figs.

## Data Availability

Data have been deposited in the repository of Universitas Syiah Kuala Research Institute.
